# Salivary levels of cariogenic bacterial species during orthodontic treatment with thermoplastic aligners or fixed appliances: a prospective cohort study

**DOI:** 10.1186/s40510-018-0230-4

**Published:** 2018-08-01

**Authors:** Iosif Sifakakis, William Papaioannou, Aikaterini Papadimitriou, Dimitrios Kloukos, Spyridon N. Papageorgiou, Theodore Eliades

**Affiliations:** 1 Department of Orthodontics, School of Dentistry, National and Kapodistrian University of Athens, Athens, Greece; 2 Department of Preventive and Community Dentistry, School of Dentistry, National and Kapodistrian University of Athens, Athens, Greece; 3 Department of Orthodontics and Dentofacial Orthopedics, 251 Hellenic Air Force General Hospital, Athens, Greece; 4 Clinic of Orthodontics and Pediatric Dentistry, Center of Dental Medicine, Faculty of Medicine, University of Zurich, Plattenstrasse 11, 8032 Zurich, Switzerland; 5 Department of Orthodontics and Dentofacial Orthopedics, School of Dental Medicine, University of Bern, Bern, Switzerland

**Keywords:** Aligners, Fixed appliances, *S*. *mutans*, *S*. *sanguinis*, *L*. *acidophilus*

## Abstract

**Background:**

Fixed orthodontic appliances might be associated with intraoral adverse effects on enamel, due to plaque accumulation and their colonization by oral microbes. At the same time, the demand for esthetic alternatives to orthodontic treatment, like thermoplastic aligners, is growing. However, thermoplastic aligners may behave differently intraorally than fixed appliances in terms of bacterial colonization and biofilm formation. Therefore, the aim of this prospective cohort study was to assess the salivary prevalence of the cariogenic bacteria *Streptococcus mutans*, *Lactobacillus acidophilus*, and *Streptococcus* sanguinis among adolescents treated orthodontically with thermoplastic aligners or fixed appliances.

**Methods:**

Thirty adolescent patients (17 girls/13 boys; mean age 13.8 years old) were assigned to treatment with either (i) self-ligating fixed appliances with nickel-titanium archwires or (ii) aligners constructed from clear transparent polyethylenterephthalat-glycol copolyester (PET-G) thermoplastic sheets. Whole stimulated saliva was collected from each patient at three time points: at baseline (before bonding and initiation of orthodontic therapy or before insertion of the thermoplastic aligners), after 2 weeks, and after 1 month. A simplified plaque index, a simplified gingival index, and the decayed, missing, and filled teeth (DMFT) index were assessed from the clinical examination of the patients. Microbiological analysis of salivary bacteria was performed by quantitative polymerase chain reaction, followed by descriptive and inferential statistics at the 5% level.

**Results:**

Although patients treated with aligners had significantly lower plaque and gingivitis scores throughout treatment compared to patients treated with fixed appliances, no significant difference could be found between the *S*. *mutans* counts of the two groups at any time through treatment (*P* > 0.05). On the other hand, patients treated with aligners had significantly lower salivary *S*. *sanguinis* counts at all time points than patients treated with fixed appliances (*P* < 0.05). Finally, almost no *L*. *acidophilus* were identified in the collected saliva samples in either of the treated samples.

**Conclusions:**

Within the limitations of this study, there were no differences in the salivary counts of *S*. *mutans* or *L*. *acidophilus* among adolescent patients treated for 1 month with thermoplastic aligners or self-ligating appliances. On the other hand, patients treated with aligners had lower salivary levels of *S*. *sanguinis* compared to those treated with self-ligating appliances.

## Background

Although fixed appliances have revolutionized contemporary orthodontic treatment, they can at the same time be considered a risk factor to the integrity of tooth enamel due to plaque accumulation and their colonization by oral microbes [1]. The placement of fixed orthodontic appliances complicates the use of standard oral hygiene procedures and causes alterations in the oral microflora by reducing pH, as well as by increasing plaque accumulation and the affinity of bacteria to metallic surfaces due to electrostatic reactions [2]. The insertion of fixed appliances creates new retentive areas that favor the local growth of streptococci, which in turn increase the levels of these organisms in saliva and around orthodontic appliances [3].

*Streptococcus mutans* (*S*. *mutans*) and *Streptococcus sobrinus* (*S*. *sobrinus*) have been identified as the main contributors in the pathogenesis of dental caries, and their presence contributes to the risk for enamel demineralization [4]. Increased levels of *S*. *mutans* and *Lactobacillus* species have also been reported to be detected in the oral cavity after bonding orthodontic attachments, and some studies have reported that there is a positive correlation between dental caries and the degree of infection with these bacterial species [5, 6].

The adhesion of cariogenic bacteria to fixed appliances might favor a treatment-induced biofilm on the tooth surface and lead to orthodontic enamel demineralizations [4]. Therefore, fixed orthodontic appliances could act as a potential risk factor for enamel demineralization [7], which has been observed even only 1 month post-insertion [8]. As far as the adhesion levels of cariogenic streptococci to various orthodontic raw materials are concerned, no difference in the adherence of *S*. *mutans* to stainless steel, ceramic, or plastic brackets has been found [9]. It seems that the material comprising the brackets does not significantly impact on the number of bacteria [10]. On the other hand, cariogenic streptococci seem to adhere significantly more to bonding adhesives than to bracket materials, with adhesion to resin-modified glass ionomers being the highest [11].

Self-ligating brackets were re-introduced in the last decades, with one of their main advantages being the elimination of the need for elastomeric ligatures with their increased plaque-retentive potential. Even though almost all other proposed advantages of self-ligating brackets have been rejected [12], a recent systematic review found that some minimal gains in terms of lower plaque accumulation might be associated with self-ligating appliances [13], even though these are not consistent through the whole observation period. On the other hand, the levels of *S*. *mutans* in whole saliva of orthodontically treated patients do not seem to be significantly different between conventional and self-ligating brackets [14]. The presence of a salivary pellicle and other bacterial species would seem to have a significant effect on the adhesion of *S*. *mutans*, reducing their numbers and further limiting any differences between types of fixed appliances [9].

In more recent years, the popularity of orthodontic treatment with thermoplastic aligners has grown due to increased demand for esthetic orthodontic appliances. Traditionally, these materials have been used extensively in the form of vacuum-formed retainers after the completion of orthodontic treatment. It has been reported that these retainers influence the adhesion of *S*. *mutans* and *Lactobacillus* spp., whose numbers of colonies increase 2 months after debonding [15]. However, evidence on the use of thermoplastic aligners as an alternative to fixed appliances is scarce. There is some evidence that recessed and sheltered areas of the aligner, such as the cusp tips and attachment dimples, harbor more biofilm than their flat surfaces [16]. A recent systematic review of the literature published up to 2014 indicated that orthodontic treatment with thermoplastic aligners might be superior in terms of periodontal health, as well as quantity and quality of plaque compared to conventional fixed appliances [17]. Additionally, a retrospective study indicated that the periodontal parameters of patients treated with thermoplastic aligners might be better than those treated with lingual fixed appliances [18]. On the other side, a recent randomized trial [19] found that although patients treated with thermoplastic aligners had initially better periodontal parameters than patients treated with conventional or self-ligating fixed appliances, ultimately appliance choice had no significant effect overall on periodontal health during treatment. However, to our knowledge, no studies have assessed the effect of orthodontic appliances on microbial colonization, which might have a direct influence on both caries and demineralization.

Therefore, this prospective comparative cohort study aimed to answer the following research question: Is there a difference in the salivary prevalence of cariogenic bacteria (*S*. *mutans*, *L*. *acidophilus*, *and S*. *sanguinis*) among 12–18-year-old adolescent patients treated orthodontically with thermoplastic aligners or fixed appliances for 1 month?

## Methods

### Study sample

The sample for this study was prospectively recruited for this study from patients presenting for treatment in the postgraduate clinic of the Departments of Orthodontics, School of Dentistry, University of Athens, and the Orthodontic Department of the 251 Air Force General Hospital, Athens, Greece, between September 2014 and July 2016. The following eligibility criteria were used to select appropriate patients to include in this study: adolescent patients aged 12–18 years old of any sex with no reported oral habits detrimental to periodontal health, including smoking, systemic diseases, or any medication affecting the oral cavity (including antibiotics) taken within the last 3 months; no teeth with active dental caries and/or missing teeth due to caries; and absence of periodontal disease. The patients’ orthodontic treatment plan did not include tooth extractions or other mechanics requiring the use of bands on molars. Ethical Board approval was obtained from both institutes prior to study initiation (S249/31.7.2014 and P076/AD6271/30.3.2017) and informed consent was obtained from all patients or their guardians.

The patients were assigned to one of the following two groups: (i) treatment with self-ligating fixed appliances and nickel-titanium (NiTi) archwires in both arches (In-Ovation R brackets and Sentalloy Wire 0.014 in.—both from GAC International, Central Islip, New York, USA) or (ii) treatment with passive aligners constructed from clear transparent polyethylenterephthalat-glycol copolyester (PET-G) thermoplastic sheets (0.75 mm in thickness, Duran®+, Scheu Dental, Iserlohn) for 1 month. Aligners were used for 1 month experimentally and the patients were afterwards treated with fixed appliances. The thermoplastic PET-G sheets were pressed over a dental stone model according to the manufacturer’s instructions, employing the Essix® Vacuum Thermoforming Machine (Dentsply Raintree Essix).

### Sample size calculation

Sample size calculation was based on a previous study [14] that reported mean log-*S*. *mutans* counts per milliliter saliva following appliance bonding of 4.57 with a standard deviation (SD) of 1.17. Assuming a 30% reduction in the *S*. *mutans* counts for aligners and a common SD, 13 patients per group would be needed to achieve power of 80% at alpha of 5% with a Student’s *t* test for independent samples. This was rounded up to 15 patients per group to account for data losses, to a total sample of 30 patients overall.

### Clinical protocol

Each patient received professional oral care and standardized hygiene instructions 3 weeks before the beginning of orthodontic treatment/insertion of the thermoplastic appliances using a typodont model, with specific attention to fixed appliance care. Additional instructions were given to brush the thermoplastic appliances once daily. The bonding procedure was performed with the direct technique using Transbond-XT (3M Unitek, Monrovia, Calif). Patients were instructed to wear the thermoplastic appliances full time, except when eating, drinking, or brushing their teeth. These appliances were replaced after 2 weeks with a new set.

All patients were asked to refrain from eating, drinking, and brushing 2 h prior to all clinical examination and saliva collection. These procedures were performed in a dental chair between 09:00 and 12:00 a.m. For each participant, the following clinical variables were assessed: the simplified plaque index (s-PlI), where the percentage of surfaces with plaque is recorded (taking into consideration four surfaces per tooth for all erupted teeth); the simplified gingival index (s-GI), where the presence or absence of gingival bleeding after gentle probing of the gingival margin is recorded at six sites around all fully erupted teeth; and the decayed, missing, and filled teeth (DMFT) index for the prevalence of caries. The indices were recorded after each saliva sample collection at each visit without the use of a plaque disclosing agent. DMFT index was recorded using criteria of the World Health Organization for permanent dentition [20]. All the clinical measurements within each one of the two experimental groups were performed by the same calibrated investigator (IS and AP).

### Sample collection and examination

Whole stimulated saliva was collected from each patient at three time points: (i) at baseline (T0), before bonding and initiation of orthodontic therapy, or before insertion of the thermoplastic aligners; (ii) after 2 weeks (T1); and after 1 month (T2). At all three time points, each patient chewed a paraffin gum for 5 min and spitted into plastic cups, while flow rate was calculated as milliliter per minute. From each patient, 1 ml of saliva was used to calculate the buffer capacity using a commercial buffer capacity test (CRT-buffer; Ivoclar, Vivadent, Liechtenstein). Collection of saliva samples was performed before any oral examination or manipulation so as not to disrupt the oral microbiota.

For the quantification of salivary cariogenic species (*S. sanguinis*, *L*. *acidophilus*, and *S*. *mutans*), 300 μl of stimulated saliva was transferred to sterile Eppendorf plastic vials adding 300 μl Tris EDTA buffer (TE buffer, 10 mM Tris-HCL, 1 mM EDTA, pH 7.6) and 300 μl 1 M NaOH solution. Samples were prepared in triplicate and kept frozen at − 80 °C until transported to the Laboratory of Microbiology, School of Dentistry, University of Athens, where they were used for the detection and quantification of salivary bacteria with quantitative polymerase chain reaction (qPCR).

### Statistical analysis

The primary outcome of this study was the salivary counts of *S*. *mutans*, while the secondary outcomes were the salivary counts of *L*. *acidophilus* and the salivary counts of *S*. *sanguinis*. The periodontal parameters (s-PII and s-GI) of all patients were also measured to assess their influence on the salivary levels of the bacteria. Data normality was assessed with graphs and tested formally with the Shapiro-Wilk test. In order to normalize skewed distributions, the s-PlI and s-GI were transformed with their square root, while microbiological counts were transformed with their fifth root. Descriptive statistics were calculated including absolute/relative frequencies for binary variables, means with SDs for normally distributed continuous variables, and medians with interquartile ranges (IQR) for non-normally distributed continuous variables. Differences between groups for normally and non-normally distributed continuous outcomes were assessed with *t* tests for independent samples and Mann-Whitney tests, respectively. Differences in the identification frequency of the bacteria at each time point were assessed with Fisher’s exact test.

Initial crude linear regression models were built with the transformed outcome as dependent variable, while experimental group (aligner or bracket) and time (T0, T1, and T2) were entered as independent variables. Subsequently, patient age, sex, and oral hygiene (through the s-PlI at T0) were added in the initial model one at a time, and if *P* ≤ 0.20, they were ultimately added to an adjusted model to account for confounders and including an interaction term of time with appliance. All analyses were run in Stata SE 14.2 (StataCorp LP, College Station, TX) with a two-sided alpha of 5% and an openly provided dataset [21].

Five patients were randomly chosen and their s-GIs re-measured by the same investigators (IS and AP) after 1 month for intra- and inter-examiner repeatability. Repeatability and agreement of the measurements were assessed with the concordance correlation coefficient [22] and the Bland and Altman [23] method.

## Results

### Clinical parameters

At T0, the thermoplastic aligner and bracket group were comparable for most characteristics, including gender, age, salivary flow rate, and DMFT (Table 1). The only exceptions were the periodontal parameters, assessed through the s-PlI and the s-GI, where both were higher in the bracket group compared to those in the aligner group, although only the latter was statistically significant (Table 2).

**Table 1:** Descriptive data of the included sample

Variable	Aligners	Brackets
Female—*n* (%)	8 (53%)	9 (60%)
Male—*n* (%)	7 (47%)	6 (40%)
Age—mean (SD)	13.9 (2.0)	13.6 (1.5)
Salivary flow rate—median (IQR)	1.2 (0.8–2.0)	1.4 (0.8–2.0)
DMFT—median (IQR)	0 (0–0)	0 (0–2.0)
s-PlI at T0—median (IQR)	24.0 (21.0–38.0)	30.0 (21.0–44.0)
s-GI T0—median (IQR)	21.0 (12.0–25.0)	31.0 (19.0–47.0)

*DMFT* decayed, missing, and filled teeth, *IQR* interquartile range, *SD* standard deviation, *s-GI* simplified gingival index, *s-PlI* simplified plaque index

**Table 2:** Plaque and gingival indices and testing with *t* test

Outcome	Aligner	Bracket	P*
	Mean (SD)	Mean (SD)	
s-PlI T0 (transformed)			
T0	5.34 (0.87)	5.55 (1.08)	0.56
T1	3.97 (1.29)	5.72 (1.24)	0.001
T2	4.80 (1.48)	6.15 (1.79)	0.03
s-GI T0 (transformed)			
T0	4.35 (0.82)	5.77 (1.56)	0.004
T1	4.23 (1.29)	5.71 (1.80)	0.01
T2	5.03 (1.66)	5.81 (1.69)	0.21

*P value for differences between experimental groups (aligner versus bracket) from *t* test

*SD* standard deviation, *s-GI* simplified gingival index, *s-PlI* simplified plaque index

Although the s-PlI was initially similar in the two groups at T0, statistically significant differences were seen between the aligner and the bracket group at T1 and T2 (Table 2). Regression analysis indicated that many factors were significantly associated with s-PlI (Table 3), including patient gender (where male patients had higher s-PlI than female patients) and initial s-PlI at T0 (where patients with initially high s-PII continued to do so). Apart from these, patients with aligners had statistically significantly lower s-PlI throughout treatment than patients with brackets (*P* < 0.001). Additionally, the interaction term of time with appliance was close to significance (*P* = 0.08), which was further explored by stratified analyses (Appendix) and indicated that s-PlI variation through time differed between aligner patients (where it tended to decrease through time) and bracket patients (where it tended to increase through time).

**Table 3:** Linear regressions with simplified plaque index or gingival index (both square root transformed) as dependent variable

			Crude model		Adjusted model	
Outcome	Factor	Group	*b* (95% CI)	*P*	*b* (95% CI)	*P*
s-PlI*	Appliance	Brackets	Referent		Referent	
		Aligners	− 1.11 (− 1.73 to − 0.48)	0.001	− 1.05 (− 1.55 to − 0.55)	< 0.001
	Age (per year)		NT		− 0.09 (− 0.23 to 0.06)	0.25
	Gender	Female	NT		Referent	
		Male			0.56 (0.01 to 1.11)	0.05
	Time	T0	Referent		Referent	
		T1	− 0.59 (− 1.14 to − 0.05)	0.03	− 0.59 (− 1.14 to − 0.05)	0.03
		T2	0.04 (− 0.65 to 0.72)	0.92	0.04 (− 0.65 to 0.72)	0.92
	s-PlI at T0		NT		0.35 (0.12 to 0.58)	0.003
s-GI^†^	Appliance	Brackets	Referent		Referent	
		Aligners	− 1.23 (− 2.01 to − 0.45)	0.002	− 1.16 (− 1.89 to − 0.43)	0.002
	Age (per year)		NT		NT	
	Gender	Female	Referent		Referent	
		Male	NT		NT	
	Time	T0	Referent		Referent	
		T1	− 0.09 (− 0.59 to 0.41)	0.72	− 0.09 (− 0.59 to 0.41)	0.72
		T2	0.36 (− 0.36 to 1.07)	0.33	0.36 (− 0.36 to 1.07)	0.33
	s-PlI at T0		NT		0.34 (0.04 to 0.64)	0.02

*The following confounders had *P* ≤ 0.20 in initial separate models and were added in the adjusted model: age, gender, and s-PlI at T0. No significant interaction was found for time with appliance type (*P* = 0.08)

^†^The following confounder had *P* ≤ 0.20 in initial separate models and was added in the adjusted model: s-PlI at T0 (age and gender had *P* > 0.20 and were not added). No significant interaction was found for time with appliance type (*P* = 0.37)

*b* unstandardized regression coefficient, *CI* confidence interval, *NT* not tested, *s-GI* simplified gingival index, *s-PlI* simplified plaque index

For the s-GI on the other side, a significant difference between the two groups was seen at T0, which tended to diminish through time (Table 2). Regression modeling indicated that aligner patients had lower s-GI scores compared to bracket patients and that patients with worse oral hygiene (judged by baseline s-PlI) had higher GI scores throughout treatment. On the other hand, no clear variation of s-GI through time was seen, nor any interaction of time with appliance.

Finally, the analysis of the repeated measurements showed excellent intra- and inter-rater agreement both with the concordance correlation coefficient (0.99 and 1.00 for intra- and inter-rater comparisons, respectively) and the Bland-Altman method (average difference [95% limits of agreement], − 1.40 [− 2.77 to − 0.03] and − 1.40 [− 2.22 to − 0.58] for intra- and inter-rater comparisons, respectively).

### Microbiological parameters

As far as qualitative changes in the measured bacteria are concerned, no differences in the identification of *S*. *mutans*, *L. acidophilus*, *and S*. *sanguinis* in the saliva of patients treated with aligners or brackets were found (Table 4). As far as quantitative microbiological parameters are concerned, these could be assessed only for *S*. *mutans* and *S. sanguinis*, as almost no *L*. *acidophilus* were identified in the collected saliva samples (Table 5).

**Table 4:** Cross-tabulation of positive organisms’ findings (binary yes/no variable) and Fisher’s exact tests

Bacteria	Aligner	Bracket	*P*
	*n*/*N* (%)	*n*/*N* (%)	
*S*. *mutans*			
Present T0	13/15 (87%)	14/15 (93%)	1.00
Present T1	12/15 (80%)	12/15 (80%)	1.00
Present T2	12/15 (80%)	14/15 (93%)	0.60
*L*. *acidophilus*			
Present T0	0/15 (0%)	0/15 (0%)	NC
Present T1	0/15 (0%)	1/15 (7%)	1.00
Present T2	0/15 (0%)	1/15 (7%)	1.00
*S*. *sanguinis*			
Present T0	15/15 (100%)	15/15 (100%)	NC
Present T1	15/15 (100%)	15/15 (100%)	NC
Present T2	15/15 (100%)	15/15 (100%)	NC

*n* patients with event of interest, *N* patients assessed, *NC* non-calculable

**Table 5:** Bacterial counts for each species (fifth root-transformed) at each time point and group with between-group testing with Mann-Whitney test (*) or *t* test for independent samples (+), according to normality of data

Bacteria		Aligner		Bracket	Test	*P*
*S*. *mutans* (transformed)	*n*	Mean (SD)	*n*	Mean (SD)		
Count at T0	15	9.44 (6.06)	15	12.20 (7.24)	+	0.27
Count at T1	15	8.60 (6.05)	15	11.02 (7.73)	+	0.35
Count at T2	15	8.87 (6.14)	15	11.09 (6.71)	+	0.35
*L*. *acidophilus* (transformed)	*n*	Median (IQR)	*n*	Median (IQR)		
Count at T0	15	0 (0–0)	15	0 (0–0)	*	1.00
Count at T1	15	0 (0–0)	15	0 (0–0)	*	0.76
Count at T2	15	0 (0–0)	15	0 (0–0)	*	0.76
*S*. *sanguinis* (transformed)	*n*	Mean (SD)	*n*	Mean (SD)		
Count at T0	15	23.93 (11.67)	15	32.05 (5.24)	+	0.02
Count at T1	15	21.32 (10.55)	15	41.08 (10.02)	+	< 0.001
Count at T2	15	22.43 (9.49)	15	34.75 (7.63)	+	0.001

*IQR* interquartile range, *NT* not tested, *SD* standard deviation

No significant difference in the salivary counts of *S*. *mutans* was found between the two groups at any time (Table 5), which was further confirmed by the regression analyses (Table 6). There was a small tendency for *S*. *mutans* counts to reduce during the period T0 to T1 (*P* = 0.04), but this faded at T2 and no different variation pattern was seen between the two groups (*P* for interaction = 0.67).

**Table 6:** Linear regressions with *S*. *sanguinis* or *S*. *mutans* counts (transformed) as dependent variable. The initial crude model coincided with the adjusted model, as no covariates were finally added

Bacteria	Factor	Group	*b* (95% CI)	*P*
*S*. *mutans*^†^	Appliance	Brackets	Referent	
		Aligners	− 2.47 (− 6.99 to 2.05)	0.28
	Age	NT		
	Gender	NT		
	Time	T0	Referent	
		T1	− 1.01 (− 1.96 to − 0.07)	0.04
		T2	− 0.84 (− 2.08 to 0.40)	0.18
	s-PlI at T0	NT		
*S*. sanguinis*	Appliance	Brackets	Referent	
		Aligners	− 13.40 (− 19.19 to − 7.62)	< 0.001
	Age	NT		
	Gender	NT		
	Time	T0	Referent	
		T1	3.21 (− 0.33 to 6.76)	0.08
		T2	0.60 (− 2.07 to 3.26)	0.66
	s-PlI at T0	NT		

*The following confounders had *P* > 0.20 in initial separate models and were not added in the adjusted model: age, gender, and s-PlI at T0. No significant interaction was found for time with appliance type (*P* = 0.11)

^†^The following confounders had *P* > 0.20 in initial separate models and were not added in the adjusted model: age, gender, and s-PlI at T0. No significant interaction was found for time with appliance type (*P* = 0.67)

*CI* confidence interval, *NT* not tested, *s-PlI* simplified plaque index, *b* unstandardized regression coefficient

The counts of *S*. *sanguinis* were significantly higher among bracket patients compared to those among aligner patients both at baseline and through orthodontic treatment (Table 5). Regression analysis indicated that aligner patients had significantly lower counts of *S*. *sanguinis* than bracket patients (Table 6). Additionally, there was a variation in the *S*. *sanguinis* counts during the observation period of T0 to T2, with a tendency to differ between aligner and bracket patients (*P* for interaction = 0.11). This was further explored by stratified analyses (Appendix) and indicated a variation pattern of *S*. *sanguinis* that was similar to that of s-PlI: the *S*. *sanguinis* counts showed a tendency to reduce through time among the aligner patients, while *S*. *sanguinis* counts tended to increase through time among the bracket patients.

## Discussion

The aim of the present prospective cohort study was to compare the salivary levels of cariogenic bacteria among adolescent patients treated with either thermoplastic aligners or fixed self-ligating appliances. The results indicated no difference in the salivary levels of *S*. *mutans* or *L*. *acidophilus*, although patients treated with thermoplastic aligners had lower salivary *S*. *sanguinis* levels than those treated with self-ligating appliances (Table 6). Oral microbiota attachment in orthodontic patients has been mainly associated with increased risk of *S*. *mutans* and lactobacilli colonization, among other species, thus initiating a series of events, which may lead to the development of demineralizations or caries [3, 8].

As far as the periodontal parameters are concerned, a statistically significant difference in both plaque scores (s-PlI) and gingivitis scores (s-GI) was found between fixed appliances and thermoplastic aligners, which favored the latter (Tables 2 and 3). This agrees with previous data indicating that teenagers treated with aligners display better compliance with oral hygiene, less plaque, and subtler gingival inflammatory reactions than those treated with fixed appliances [24]. The ease of oral hygiene maintenance with the clear aligners most likely allows patients to maintain, or possibly even improve, their oral hygiene. A recent systematic review pointed out that periodontal health indexes are significantly improved during clear aligner treatment, in particular when these appliances were compared to fixed appliances. However, the level of evidence was moderate for all the included studies [17]. Additionally, oral hygiene was significantly associated with patient sex, with male patients having significantly higher plaque scores than female patients (Table 3). Furthermore, pre-treatment oral hygiene levels were significantly associated with plaque scores and gingivitis during treatment (Table 3). Finally, no clear variation pattern of oral hygiene was seen through time, which agrees with Clements et al. [25], who demonstrated that the mean average papillary bleeding scores did not change in a statistically significant manner during aligner treatment.

In the present study, instructions were given to brush the thermoplastic appliances once daily. However, a recent study demonstrated that the use of a vibrating bath with cleaning solution protocol reduced biofilm adherence more than regular brushing or immersion of the aligner in chlorhexidine mouthwash [26]. The use of a chlorhexidine mouthwash as an adjunct to oral hygiene at home does not seem to be necessary for patients undergoing aligner treatment, at least for the first 8 months of treatment [27].

Additionally, several appliance-related factors might influence the intraoral performance of thermoplastic aligners. The material used for the fabrication of the thermoplastic aligners in this study was PET-G, which is the most widely used material for the fabrication of both aligners and retainers [28]. The material used for the Invisalign (Align Technology, Santa Clara, Calif) aligners is polyurethane-based and seems to have higher hardness and modulus values, a slightly higher brittleness, and lesser creep resistance compared to PETG-based products [28]. However, no evidence on their microbiological colonization exists to our knowledge. It has been suggested that the surface morphology of the aligner might contribute to bacterial adhesion and thereby to salivary bacteria levels. The surface of aligners is not completely smooth but exhibits microabrasions and irregularities, and this configuration with its furrowed corrugated facade makes the appliance more conducive to bacterial and biofilm accumulation [16].

Furthermore, the gingival coverage of an aligner, which differs across the various systems, might directly influence periodontal parameters and microbial colonization. Although Invisalign aligners have no significant gingival coverage, other aligner systems are trimmed to overlap the attached gingiva, in order to improve retention. This method is claimed to provide improved aligner retention, which might however come at the cost of periodontal implications. Furthermore, the manufacturing process may also play an important role in the aligner’s surface, as pressure-forming involves higher pressures than vacuum-forming, which might affect up to a limit the detail of the inner, fitting surface of the aligner [29]. The aligners used in the present study were vacuum-formed and cut 2 mm higher than the gingival margin.

Finally, the use of bonded attachments during treatment with thermoplastic aligners might provide additional plaque retentive surfaces on the patient’s teeth and thereby increase the intraoral microbial load. However, no such bonded attachments were used in any patients of the present study and therefore the results of this study might not fully reflect cases where multiple irregular attachments are bonded on the teeth to improve the aligner efficacy [30].

Even though the present study provides up to now missing evidence on the microbiological performance of orthodontic thermoplastic aligners, it also has some limitations. It is important to note that patients in the present study were followed for a short term of 1 month. Another study evaluating the effects of fixed appliances over a longer period indicated that both periodontal health and subgingival plaque composition deteriorated from appliance insertion to the first 3 months but then improved during the subsequent 3 months [31]. A similar finding was seen by Karkhanechi et al. [32] who found an initial deterioration of periodontal parameters after fixed appliance insertion that improved after 6 months of treatment. Additionally, a recent randomized trial [19] found that although aligner patients tended to have better plaque and gingival bleeding scores than conventional or self-ligating fixed appliance patients in the short term, no difference could be found for the whole treatment duration. Therefore, it might well be that the short-term salivary levels of cariogenic bacteria observed in this study might not reflect the long-term results. Moreover, only adolescent patients were included in this study and therefore its results might not be generalizable to adult patients. Finally, the present prospective study was not randomized and might be prone to some bias [33].

## Conclusions

Within the limitations of the present short-term prospective study, no differences could be found in the salivary levels of *S*. *mutans* and *L*. *acidophilus* between adolescent patients treated for 1 month with thermoplastic aligners or self-ligating appliances. On the other hand, lower salivary levels of *S*. *sanguinis* were found in patients treated with thermoplastic aligners compared to those treated with self-ligating fixed appliances.

## Appendix

**Table 7:** Explorative regression analyses with simplified plaque index or *S*. *sanguinis* counts as dependent variable and stratified by appliance subgroup

			Aligners		Brackets		
Outcome	Factor	Group	*b* (95% CI)	*P*_SG_	*b* (95% CI)	*P*_SG_	*P*_interaction_
s-PlI	Age	Per year	− 0.06 (− 0.23 to 0.11)	0.47	− 0.18 (− 0.43 to 0.07)	0.16	0.08
	Gender	Female	Referent		Referent		
		Male	0.25 (−0.61 to 1.10)	0.57	0.95 (0.23 to 1.67)	0.01	
	Time	T0	Referent		Referent		
		T1	− 1.37 (− 2.04 to − 0.69)	< 0.001	0.18 (− 0.48 to 0.84)	0.60	
		T2	− 0.54 (− 1.29 to 0.21)	0.16	0.61 (− 0.49 to 1.71)	0.28	
	s-PlI at T0	Per unit	0.53 (0.16 to 0.89)	0.005	0.20 (− 0.13 to 0.54)	0.24	
*S*. *sanguinis*	Age	Per year	NT		NT		0.11
	Gender	Female	NT		NT		
		Male	NT		NT		
	Time	T0	Referent		Referent		
		T1	− 2.61 (− 4.89 to − 0.33)	0.03	9.03 (3.71 to 14.35)	0.001	
		T2	− 1.50 (− 4.47 to 1.46)	0.32	2.70 (− 1.58 to 6.97)	0.22	
	s-PlI at T0	per unit	NT		NT		

*b* unstandardized regression coefficient, *CI* confidence interval, *NT* not tested, *P*_*interaction*_ *P* value for time differences between appliance subgroups (interaction time with appliance), *P*_*SG*_ *P* for effects within each subgroup, *s-PlI* simplified plaque index
